# Germinated Soybean Embryo Extract Ameliorates Fatty Liver Injury in High-Fat Diet-Fed Obese Mice

**DOI:** 10.3390/ph13110380

**Published:** 2020-11-11

**Authors:** Doyoung Kwon, Sou Hyun Kim, Seung Won Son, Jinuk Seo, Tae Bin Jeong, Kyung-Mi Kim, Jae-Chul Jung, Mi Sook Jung, Yun-Hee Lee, Young-Suk Jung

**Affiliations:** 1 Lab of Molecular Toxicology, College of Pharmacy, Pusan National University, Busan 46241, Korea; doyoung.kwon@pusan.ac.kr (D.K.); souhyun@pusan.ac.kr (S.H.K.); wellsu123@pusan.ac.kr (S.W.S.); hanabuddy@pusan.ac.kr (J.S.); 2 Life Science Research Institute, Novarex Co., Ltd., Cheongju 28126, Korea; jtaebin@novarex.co.kr (T.B.J.); kkm3507@novarex.co.kr (K.-M.K.); jcjung@novarex.co.kr (J.-C.J.); 3 Biotoxtech Co., Ltd. Ochang Scientific Industrial Complex, 53, Yeongudanji-ro, Cheongju 28115, Korea; mszheng@biotoxtech.com; 4 College of Pharmacy and Research Institute of Pharmaceutical Sciences, Seoul National University, Seoul 08826, Korea

**Keywords:** germinated soybean embryo, high-fat diet, obesity, non-alcoholic fatty liver disease, lipid metabolism, adipose tissue, inflammation

## Abstract

Soybean is known to have diverse beneficial effects against human diseases, including obesity and its related metabolic disorders. Germinated soybean embryos are enriched with bioactive phytochemicals and known to inhibit diet-induced obesity in mice, but their effect on non-alcoholic fatty liver disease (NAFLD) remains unknown. Here, we germinated soybean embryos for 24 h, and their ethanolic extract (GSEE, 15 and 45 mg/kg) was administered daily to mice fed with a high-fat diet (HFD) for 10 weeks. HFD significantly increased the weight of the body, liver and adipose tissue, as well as serum lipid markers, but soyasaponin Ab-rich GSEE alleviated these changes. Hepatic injury and triglyceride accumulation in HFD-fed mice were attenuated by GSEE via decreased lipid synthesis (SREBP1c) and increased fatty acid oxidation (*p*-AMPKα, PPARα, PGC1α, and ACOX) and lipid export (MTTP and ApoB). HFD-induced inflammation (TNF-α, IL-6, IL-1β, CD14, F4/80, iNOS, and COX2) was normalized by GSEE in mice livers. In adipose tissue, GSEE downregulated white adipose tissue (WAT) differentiation and lipogenesis (PPARγ, C/EBPα, and FAS) and induced browning genes (PGC1α, PRDM16, CIDEA, and UCP1), which could also beneficially affect the liver via lowering adipose tissue-related circulating lipid levels. Thus, our results suggest that GSEE can prevent HFD-induced NAFLD via inhibition of hepatic inflammation and restoration of lipid metabolisms in both liver and adipose tissue.

## 1. Introduction

Obesity, which is caused by overnutrition and low energy expenditure, is an important risk factor for various human diseases. Non-alcoholic fatty liver disease (NAFLD), one of the obesity-related metabolic disorders which are usually caused by high-calorie and high-fat diets (HFD), is a growing health issue worldwide [1,2]. Similarly to alcoholic fatty liver disease, the histopathological spectrum of NAFLD ranges from simple hepatic lipid accumulation (steatosis) to inflammatory steatohepatitis with fibrosis, which can further progress to cirrhosis and cancer [1,2]. Hepatic injury and dysfunction that occur in NAFLD are closely associated with the development of other metabolic diseases, including type 2 diabetes, hypertension, and dyslipidemia [1,2]. However, there is currently no FDA-approved medication for the treatment of NAFLD. 

Dietary soybean (*Glycine max* (L.) *Merr.*) has gained growing interests due to its high protein content and beneficial effects against chronic human diseases, such as obesity, cardiovascular diseases, type II diabetes, renal disease, and cancers [3,4,5]. Many clinical trials and animal studies have demonstrated that soy-related products, such as soy extracts, isolated soy proteins, and their compound ingredients, can reduce body and adipose tissue weight and improve serum lipid biomarker levels, suggesting their potent anti-obesity effects [6,7,8,9]. Moreover, dietary soybean has been shown to have inhibitory effects against HFD-induced hepatic steatosis in animal studies [10,11,12,13]. 

Previous studies have suggested that the main bioactive components of soybean are soyasaponins and isoflavones [4,14]. Moreover, soyasaponins and polyphenolic isoflavones, such as genistein, daidzein, and glycitein, have been reported to prevent alcoholic/non-alcoholic liver diseases and/or other liver diseases, including hepatitis and tumors, due to their hypolipidemic, anti-inflammatory, antioxidant, and anti-cancer properties [8,15,16,17,18,19].

The soybean embryo is a small part (2% of the total weight) of the whole soybean but is known to contain concentrations of soyasaponins and isoflavones, which are 3.1- and 7.5-times higher, respectively, than those found in other parts of the soybean, such as the cotyledon [20,21,22,23]. Notably, the dietary intake of soybean embryo powder was shown to inhibit obesity and NAFLD in HFD-fed mice [24]. Recently, it was found that germination of the soybean embryo increases soyasaponin Ab and total isoflavone contents when compared to those found in the embryo before germination [25,26]. Moreover, an extract of the germinated soybean embryo was shown to effectively reduce body weight gain and adipose tissue size in an obese mouse model by upregulating lipolysis and β-oxidation in the adipose tissue and promoting white adipose tissue (WAT) browning [27]. However, the effects of the phytochemical-rich germinated soybean embryo extract on NAFLD remains unknown.

In the present study, we prepared an ethanolic soybean embryo extract after 24 h of germination, which contained a high concentration of soyasaponin Ab. Then, the extract was administered to HFD-fed obese mice to determine its effect on hepatic steatosis. The results showed that the germinated soybean embryo extract (GSEE) significantly attenuated HFD-induced NAFLD and obesity in the mice by improving lipid metabolism in both the liver and adipose tissue, as well as inhibiting hepatic inflammation. 

## 2. Results

### 2.1. Concentration of Soyasaponin Ab in the GSEE 

**Figure 1:**
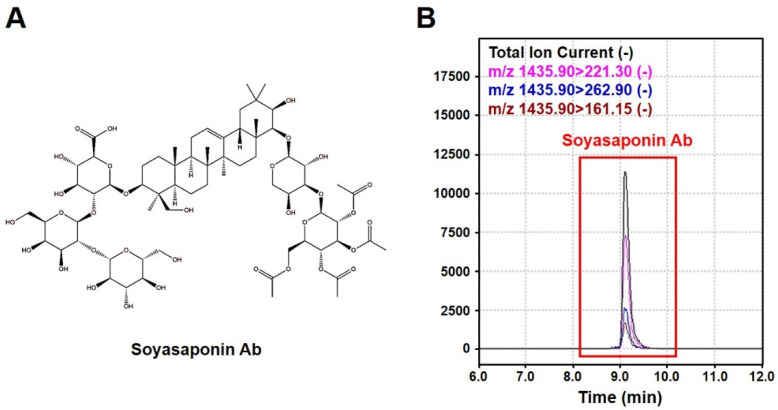
Analysis of soyasaponin Ab contents in the germinated soybean embryo extract (GSEE) using HPLC/MS/MS: (**A**) chemical structure of soyasaponin Ab; (**B**) HPLC chromatogram of soyasaponin Ab in the GSEE.

The embryos of soybeans (*Glycine max* (L.) *Merr.*) separated from the seed coats and cotyledons were germinated for 24 h, and the extract was obtained using 50% ethanol as described in the Materials and Methods. To confirm the nutritional enrichment of the soy embryo by germination, we analyzed one of the main bioactive compounds, soyasaponin Ab, by using an HPLC/MS/MS system (Figure 1), and found that its concentration in the germinated soybean embryo was 77.0 ± 1.3 mg/g. In a previous study, soyasaponin Ab contents in the soybean cotyledon and embryo were reported to be 0.32–0.40 mg/g and 12.98–27.87 mg/g, respectively [20]. Thus, the germinated soy embryo used in our study appears to contain a much higher concentration of soyasaponin Ab than other soybean parts, as well as the embryo before the germination.

### 2.2. Anti-Obesity and Blood Lipid Lowering Effects of the GSEE in HFD-Fed Mice

**Figure 2:**
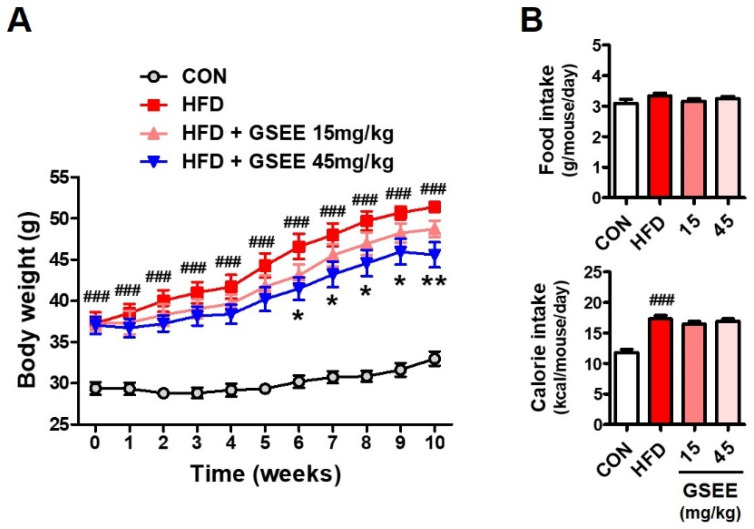
Effects of GSEE on the body weight in high-fat diet (HFD)-fed obese mice; (**A**) body weight changes; (**B**) food and calorie intake. Mice (*n* = 8 in each group) were fed with HFD for 10 weeks with or without daily oral administration of GSEE (15 or 45 mg/kg of body weight). Values are represented as mean ± SE. Student’s *t*-test, ### *p* < 0.001 vs. control. *, ** *p* < 0.05, and 0.01, respectively, vs. HFD.

**Figure 3:**
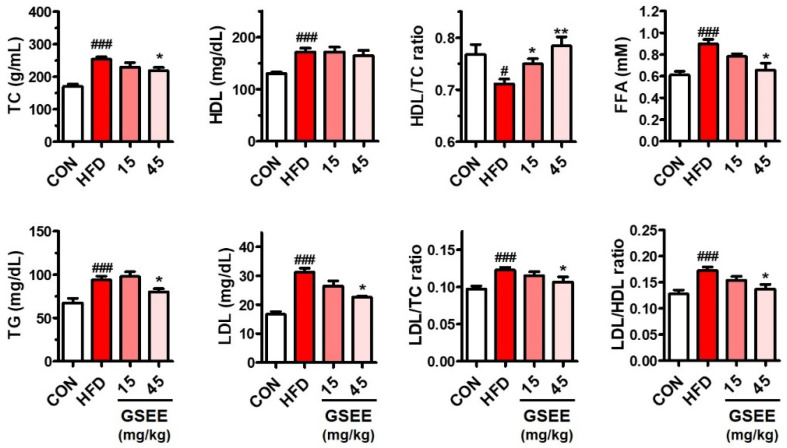
Effects of the GSEE on serum lipid markers in HFD-fed obese mice. Mice (*n* = 8 in each group) were fed with HFD for 10 weeks with or without daily oral administration of GSEE (15 or 45 mg/kg of body weight). Values are represented as mean ± SE. Student’s *t*-test, ### *p* < 0.001 vs. control. *, ** *p* < 0.05, and 0.01, respectively, vs. HFD. TC, total cholesterol; TG, triglyceride; HDL; high-density lipoproteins; LDL, low-density lipoproteins; FFA, free fatty acids.

The anti-obesity effect of the GSEE was determined by using an HFD-induced obesity mouse model. Male C57BL/6J mice (13-week-old) fed with HFD (60% of calories from fat) were orally administered with GSEE (0, 15, or 45 mg/10 mL/kg of body weight) once a day for 10 weeks, and compared with the control mice provided with normal diet (10% of calories from fat). The amount of daily food intake was not different, but the total calorie intake was higher in the mice fed HFD (Figure 2B) because of the different energy densities of the rodent chows (control, 3.82 kcal/g; HFD, 5.21 kcal/g). GSEE treatment did not affect the food and calorie intake of HFD-fed mice (Figure 2B). Mice in the HFD group were shown to have a 56% higher body weight than control mice at the end of the experiment (Figure 2A). However, treatment with the GSEE (45 mg/kg) lowered the HFD-induced body weight gain by 32.6% without any abnormal clinical symptoms. Since GSEE has been shown to be safe to animals and cultured cells in previous studies [26,27], the decrease in body weight by GSEE in our study was considered as the anti-obesity effect, not an adverse effect of this extract. The levels of serum lipid-related markers, such as total cholesterol (TC), triglyceride (TG), high-density lipoproteins (HDL), low-density lipoproteins (LDL), and free fatty acids (FFA), as well as LDL/TC and LDL/HDL ratios, were also shown to be elevated by HFD (Figure 3). However, GSEE administration (45 mg/kg) significantly reduced the levels of all previously mentioned markers, with the exception of HDL (Figure 3). The decreased HDL/TC ratio in the HFD group was also normalized by GSEE treatment (45 mg/kg) (Figure 3). These results indicate that GSEE suppresses HFD-induced obesity and improves the circulating lipid status in mice.

### 2.3. Effects of GSEE on HFD-Induced Liver Weight Changes and Hepatocellular Injury in Mice

**Figure 4:**
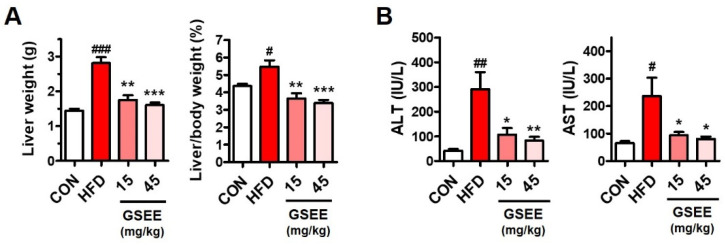
Effects of GSEE on liver weight and serum hepatotoxicity indicators in HFD-fed obese mice: (**A**) liver weight and the ratio between liver and body weight (%); (**B**) serum alanine transaminase (ALT) and aspartate transaminase (AST) activities. Mice (*n* = 8 in each group) were fed with HFD for 10 weeks with or without daily oral administration of GSEE (15 or 45 mg/kg of body weight). Values are represented as mean ± SE. Student’s *t*-test, #, ##, ### *p* < 0.05, 0.01, and 0.001, respectively, vs. control. *, **, *** *p* < 0.05, 0.01, and 0.001, respectively, vs. HFD.

To examine the hepatic effects of GSEE, liver weight and serum hepatotoxicity markers were measured and compared between the groups. HFD significantly elevated not only the liver weight but also the liver/body weight ratio (%) in the mice (Figure 4A). GSEE administration (15 or 45 mg/kg) in HFD mice was found to markedly reduce their liver weight to values similar with those of control mice (Figure 4A). Interestingly, 15 mg/kg of GSEE did not alter the body weight (Figure 2) but lowered the liver weight and the ratio between the liver and body weight, suggesting that GSEE has a specific effect on mouse livers. Serum hepatotoxicity indicators, alanine transaminase (ALT) and aspartate transaminase (AST) levels, which were highly elevated by HFD, were normalized by treatment with both doses of GSEE (15 and 45 mg/kg). This indicates that GSEE has a potent hepatoprotective effect (Figure 4B).

### 2.4. Inhibition of Hepatic Steatosis by GSEE in Mice

**Figure 5:**
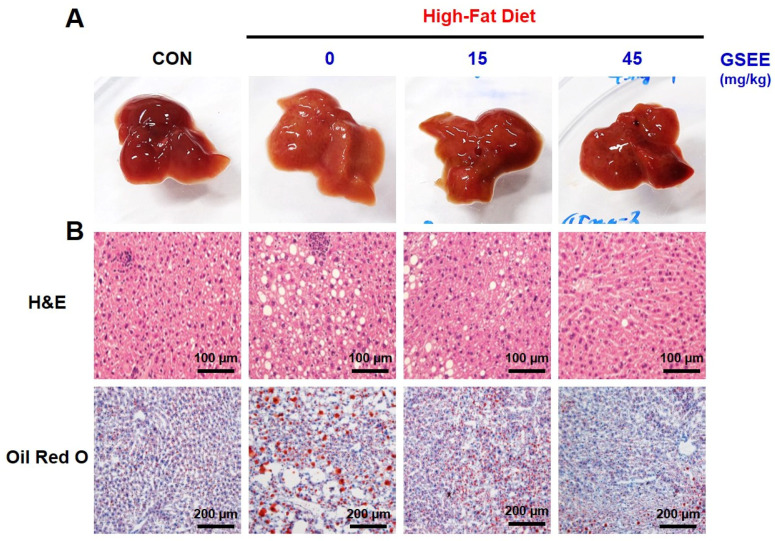

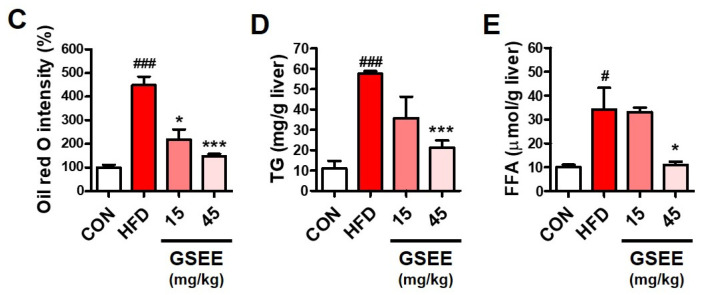
Inhibitory effects of GSEE on HFD-induced hepatic steatosis in mice: (**A**) liver tissues; (**B**) microscopic images of hematoxylin & eosin (H&E)- and oil red O-stained liver tissues; (**C**) relative oil red O intensity; (**D**) hepatic triglyceride (TG) levels; (**E**) hepatic free fatty acid (FFA) levels. Mice (*n* = 8 in each group) were fed with HFD for 10 weeks with or without daily oral administration of GSEE (15 or 45 mg/kg of body weight). Relative oil red O intensity was measured using microscopic images of liver tissues from randomly selected 3 mice in each group. Values are represented as mean ± SE. Student’s *t*-test, #, ### *p* < 0.05 and 0.001, respectively, vs. control. *, *** *p* < 0.05 and 0.001, respectively, vs. HFD.

Hepatic lipid accumulation, a hallmark of NAFLD, was determined to investigate the anti-steatogenic effect of GSEE. HFD-fed mice showed more yellowish livers compared to control mice, but GSEE reversed the liver color to dark brown (Figure 5A). The microscopic images of liver tissues stained with hematoxylin & eosin (H&E) and oil red O show that HFD induced a significant accumulation of lipid droplets (Figure 5B). The hepatic TG level in the HFD group (57.5 mg/g liver), which indicates steatosis, was found to be 6-fold higher than that of the control group (Figure 5D). However, GSEE treatment reduced the number of lipid droplets (Figure 5B) as well as the relative intensity of oil red O (Figure 5C) in a dose-dependent manner. Moreover, treatment with 45 mg/kg of GSEE decreased TG levels by 63.2% when compared to the HFD only group (Figure 5D). Hepatic FFA levels were also elevated by HFD and decreased by GSEE treatment (45 mg/kg) (Figure 5E).

### 2.5. GSEE Restores the Dysregulated Hepatic Lipid Metabolism in HFD-Fed Mice 

**Figure 6:**
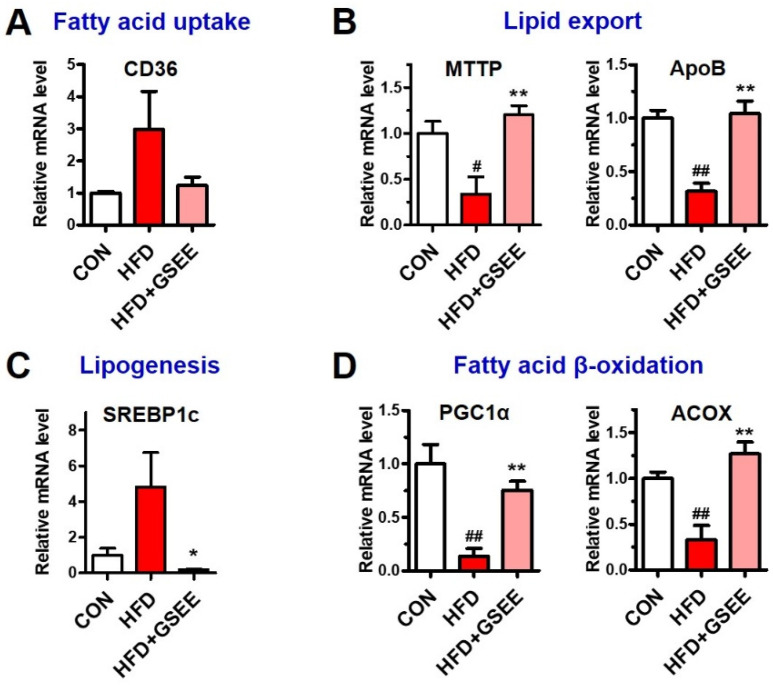
Effects of GSEE on the expression of genes involved in lipid metabolism in HFD-fed mouse livers: (**A**) relative CD36 mRNA levels representing fatty acid uptake; (**B**) microsomal triglyceride transfer protein (MTTP) and apolipoprotein B (ApoB) mRNA levels representing lipid export; (**C**) sterol regulatory element-binding protein-1c (SREBP1c) mRNA level representing de novo lipogenesis; (**D**) peroxisome proliferator-activated receptor-γ, coactivator-1α (PGC1α) and acyl-CoA oxidase (ACOX) mRNA levels representing fatty acid β-oxidation. Mice were fed with HFD for 10 weeks with or without daily oral administration of GSEE (45 mg/kg of body weight). Liver samples from randomly selected 4 mice in each group were used for qRT-PCR. Values are represented as mean ± SE. Student’s *t*-test, #, ## *p* < 0.05 and 0.01, respectively, vs. control. *, ** *p* < 0.05, and 0.01, respectively, vs. HFD.

**Figure 7:**
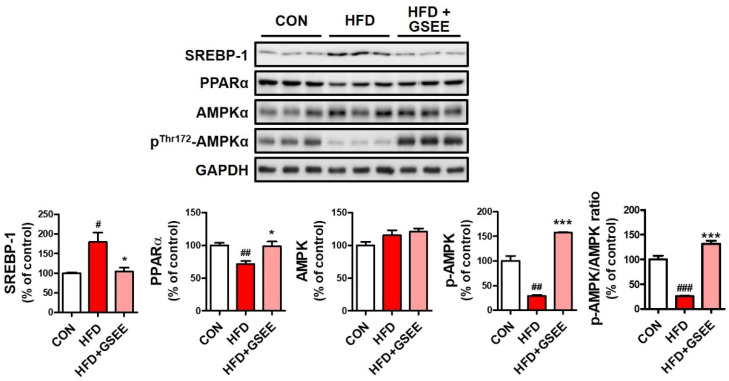
Effects of GSEE on the hepatic protein levels involved in lipid metabolism in HFD-fed mice: mice were fed with HFD for 10 weeks with or without daily oral administration of GSEE (45 mg/kg of body weight). Liver samples from randomly selected 3 mice in each group were used for Western blot analysis. Values are represented as mean ± SE. Student’s *t*-test, #, ##, ### *p* < 0.05, 0.01, and 0.001, respectively, vs. control. *, *** *p* < 0.05 and 0.001, respectively, vs. HFD.

To identify the hepatic mechanisms underlying the anti-steatogenic effect of GSEE, we determined the mRNA expressions and protein levels involved in fatty acid uptake, lipid transport, de novo lipogenesis, and fatty acid β-oxidation in the liver. The expression of cluster of differentiation 36 (CD36), which is a long-chain fatty acid translocase located at the plasma membrane of hepatocytes and plays a role in fatty acid uptake, was found to be increased in the HFD group but was not significantly higher than that of other groups (Figure 6A). The expressions of microsomal triglyceride transfer protein (MTTP) and apolipoprotein B (ApoB), which are proteins involved in hepatic lipid export via very-low-density lipoprotein (VLDL) synthesis [1], were found to be significantly downregulated by HFD. Previous studies have shown that NAFLD patients with simple steatosis exhibit higher hepatic expression of MTTP and ApoB, as well as higher serum VLDL levels. However, other studies have shown that their expression was significantly reduced in non-alcoholic steatohepatitis (NASH) patients when compared with healthy individuals [28,29,30]. Thus, the decrease in MTTP and ApoB expression found in our study (Figure 6B) suggests the severe progression of fatty liver disease. Moreover, the fact that GSEE treatment recovered their expressions (Figure 6B) suggests that the extract plays a regulatory role in hepatic lipid export. The protein level and mRNA expression of a key transcription factor for de novo lipogenesis, sterol regulatory element-binding protein-1c (SREBP1c) [31], were increased in HFD-fed mice but markedly decreased by GSEE treatment (Figure 6C and Figure 7). Adenosine monophosphate-activated protein kinase (AMPK), a serine/threonine protein kinase, is known to inhibit lipogenesis by inhibiting acetyl-CoA carboxylase (ACC) activity [32]. The total AMPK protein level did not show any difference between the groups (Figure 7). However, the decreased level of activated AMPK (phosphorylated at threonine-172) in the liver of HFD-fed mice was significantly prevented by GSEE treatment (Figure 7), suggesting the inhibitory effect of GSEE on HFD-induced hepatic lipid synthesis. Hepatic fatty acid β-oxidation also appeared to be inhibited in HFD mice, as shown by the decrease in peroxisome proliferator-activated receptor-γ, coactivator-1α (PGC1α) and acyl-CoA oxidase (ACOX) expression (Figure 6D). PGC1α, the key regulator of mitochondria biogenesis and fatty acid β-oxidation, induces the expression of ACOX that catalyzes the first step of the peroxisomal fatty acid oxidation process [1,33]. Peroxisome proliferator-activated receptor-α (PPARα), the predominant form of PPARs in liver, is a transcription factor that regulates fatty acid catabolism and clearance [34]. ACOX is also a target of PPARα, and the downregulation of PPARα has been found in NAFLD patients [34]. In the present study, the decreased PGC1α and ACOX mRNA levels and PPARα protein by HFD were normalized by GSEE (Figure 6D and Figure 7). Thus, these results indicate that GSEE activates peroxisomal and mitochondrial fatty acid oxidation. Since AMPK is known to phosphorylate PGC1α [32], which also increases MTTP and ApoB expression [35], our results suggest that GSEE could exert its anti-steatosis effect by regulating multiple processes such as lipid export, synthesis, and consumption in mouse livers.

### 2.6. Anti-Inflammatory Effect of GSEE in HFD-Fed Mouse Livers

**Figure 8:**
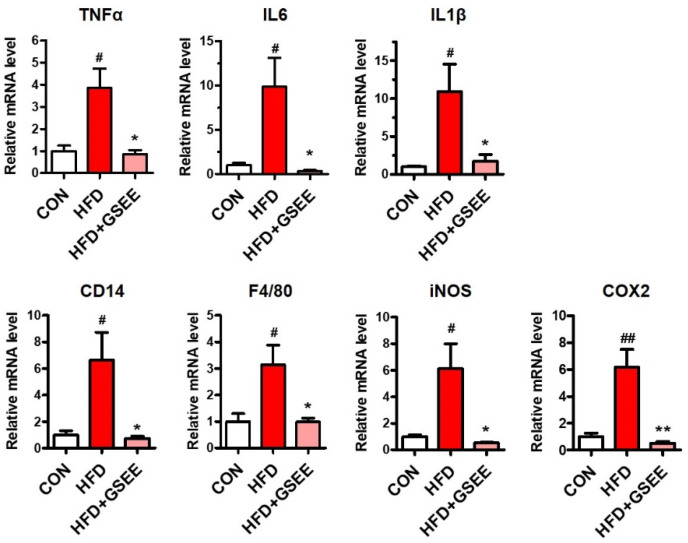
Suppressive effects of GSEE on the expression of inflammatory genes in HFD-fed mouse livers. Mice were fed with HFD for 10 weeks with or without daily oral administration of GSEE (45 mg/kg of body weight). Liver samples from randomly selected 4 mice in each group were used for qRT-PCR. The values are expressed as mean ± SE. Student’s *t*-test, #, ## *p* < 0.05 and 0.01, respectively, vs. control. *, ** *p* < 0.05, and 0.01, respectively, vs. HFD.

Significant hepatic inflammation has been observed in chronic NAFLD, which is usually referred to as NASH [36]. Lipid accumulation stimulates hepatic inflammatory cells and hepatocytes to secrete inflammatory cytokines and mediators, such as tumor necrosis factor-α (TNFα), interleukin (IL)-6, IL-1β, inducible nitric oxide synthase (iNOS), and cyclooxygenase-2 (COX2), which is usually correlated with the severity of NAFLD [36,37]. In the present study, the hepatic mRNA levels of these inflammatory mediators were increased by HFD but significantly inhibited by GSEE treatment (Figure 8). Activated Kupffer cells play central role in the inflammatory progression of NAFLD via cytokine secretion, and cluster of differentiation 14 (CD14), a membrane component of Kupffer cells, was found to be elevated in NASH patients [38,39,40]. The expressions of CD14 and F4/80, markers of Kupffer cells and their phagocytic function, were induced by HFD but normalized by GSEE (Figure 8). Inflammatory cytokines, such as TNF-α, can be cytotoxic by themselves and induce insulin resistance and apoptosis [36,41]. It has been also reported that iNOS induction in NAFLD leads to *S*-nitrosylation of the insulin receptor, thereby causing insulin resistance [37]. Moreover, COX2 was shown to interact with TNF-α and IL-6 and induce hepatocellular apoptosis in a rat NASH model [42]. Therefore, the GSEE-induced attenuation of the inflammatory responses in HFD-fed mouse livers can be a preventative mechanism for hepatic injuries.

### 2.7. GSEE Inhibits White Adipocyte Differentiation but Stimulates Brown Adipocyte Differentiation in HFD-Fed Mouse Livers 

**Figure 9:**
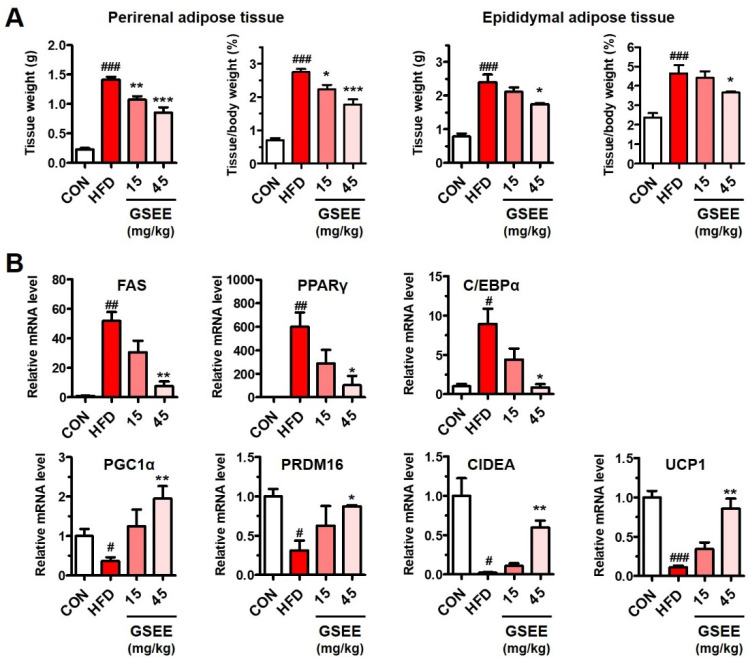
Effects of the GSEE on the adipose tissue weights, and the gene expressions involved in lipid metabolism and adipocyte differentiation in HFD-fed obese mice: (**A**) perirenal and epididymal adipose tissue weights and the ratios to body weight (%); (**B**) relative mRNA expressions. Mice (*n* = 8 in each group) were fed with HFD for 10 weeks with or without daily oral administration of GSEE (15 or 45 mg/kg of body weight). Adipose tissues from randomly selected 3 mice in each group were used for qRT-PCR. Values are represented as mean ± SE. Student’s *t*-test, #, ##, ### *p* < 0.05, 0.01, and 0.001, respectively vs. control. *, **, *** *p* < 0.05, 0.01, and 0.001, respectively, vs. HFD.

Adipose tissues, the main regulatory site of energy by storing fat or secreting FFA, can critically affect the lipid metabolism of liver [43,44]. Thus, we examined the influence of GSEE on the adipose tissues to identify the association with the inhibition of hepatic lipid accumulation. The perirenal and epididymal adipose tissue weights were increased 4.6- and 2.8-fold by HFD, respectively, while GSEE treatment (45 mg/kg) reduced those weights in a dose-dependent manner (Figure 9A). To determine the mechanisms underlying the adipose tissue-reducing effects of GSEE, we examined mRNA levels of genes involved in the lipid metabolism of the WAT. The adipose tissues obtained from HFD-fed mice showed a significant increase in the mRNA levels of fatty acid synthetase synthase (FAS), PPARγ, and CCAAT-enhancer-binding protein-α (C/EBPα) (Figure 9B), indicating an increase in lipid synthesis, fatty acid storage, and white adipocyte differentiation, respectively [45]. However, GSEE administration lowered the mRNA levels of these genes in a concentration-dependent manner (Figure 9B). In contrast, the HFD-induced decreased expression of the PGC1α, PR domain containing 16 (PRDM16), cell death-inducing DNA fragmentation factor-like effector A (CIDEA), and uncoupling protein 1 (UCP1) was increased by GSEE treatment (Figure 9B). PPARγ, which is an essential transcription factor for adipocyte differentiation, has been found to be induced during adipogenesis [46]. White adipocyte differentiation requires C/EBPα, but brown adipocyte differentiation is not dependent on this transcription factor [47]. PRDM16 is enriched in brown fat and upregulates thermogenic genes, such as CIDEA and UCP1 [48]. CIDEA, a lipid droplet-associated protein that enhances UCP1 transcription, is highly expressed in brown adipocytes but not found in mouse WAT [49]. UCP1, a transmembrane protein found in brown adipocyte mitochondria, uncouples the respiratory chain resulting in heat generation rather than ATP production [50]. Thus, the results of the present study indicate that GSEE treatment inhibited white adipocyte differentiation but stimulated brown adipocyte differentiation in HFD-fed mice. GSEE appears to mitigate adiposity by reducing lipogenesis and fat storage, as well as inducing fatty acid oxidation and thermogenic energy consumption in the adipose tissue. The lipid-lowering effect of GSEE in both the blood (Figure 3) and liver (Figure 5) could be closely related with the alterations in the adipose tissue (Figure 9).

## 3. Discussion

In the present study, we demonstrated that the soyasaponin-rich GSEE significantly attenuated HFD-induced hepatic steatosis as well as obesity in mice. Increases in the amount of circulating lipids from dietary sources can adversely affect blood vessels and induce abnormal lipid accumulation in peripheral tissues [51]. The recovery of serum lipid levels after GSEE treatment and the increase in the ratio between good cholesterol (HDL) and total cholesterol (Figure 3) could beneficially affect the lipid metabolism in the adipose tissue and liver, thereby resulting in reduced obesity and tissue fat accumulation in mice.

The liver is an important organ that regulates systemic lipid metabolism via the uptake of FFA, as well as synthesizing, storing, and exporting lipids [52]. Hepatic delivery of circulating FFA can impair the insulin sensitivity of this organ, which induces SREBP-1c transcription and leads to hepatic de novo lipogenesis [44]. FFA act as pro-inflammatory molecules by stimulating the hepatic NF-κB pathway and activating Kupffer cells which secrete TNF-α [44,53]. FFA and TNF-α are known to be cytotoxic [36,41,44]. Thus, in the present study, the normalization of serum and hepatic FFA levels by GSEE treatment could explain the reduction in lipogenesis, inflammation, and hepatocellular injury. Improvement in hepatic steatosis by treatment with soy-related products and the cellular mechanisms underlying these improvements have been reported in many studies [6,8]. Soy isoflavones prevent obesity and hepatic steatosis by inhibiting mTORC1 via Akt phosphorylation, which results in the downregulation of SREBP1c and the upregulation of PPARα [54]. Isoflavone-fed rats were also shown to exhibit inhibited hepatic lipogenesis and enhanced lipolysis and β-oxidation [54]. In our study, the increase in PGC1α and ACOX expression (Figure 6D), and PPARα protein (Figure 7) by GSEE indicates the stimulation of mitochondrial and peroxisomal fatty acid β-oxidation, which could increase the catabolism of FFA and prevent de novo lipogenesis and lipotoxicity. It was reported that hepatic AMPK is activated by a soy-containing diet in mice [55]. Moreover, the reduced expression of AMPK in high-fat/cholesterol diet-fed mouse livers was restored by soy embryo administration [24]. Inactivation of ACC by AMPK reduces the synthesis of malonyl-CoA, an inhibitor of carnitine palmitoyltransferase-1 (CPT1) [32]. Thus, the activation of AMPK consequently promotes the CPT1-mediated fatty acid uptake into mitochondria for β-oxidation [32]. Therefore, the increased phosphorylation at Thr172 of AMPK by GSEE (Figure 7) might have dual anti-steatogenic effects on the livers, the inhibition of fatty acid synthesis and the stimulation of fatty acid oxidation, and, thus, the AMPK activation can be an important mechanism of the anti-steatogenic action of GSEE. Decreased hepatic lipid export with reduced MTTP and ApoB expression in severe NAFLD is one of the causes of aggravated hepatic lipid accumulation [1]. Therefore, the restoration of MTTP and ApoB mRNA levels by GSEE treatment (Figure 6B) also can also act as an inhibitory mechanism of HFD-caused steatosis. In many studies, soyasaponins have been reported to reduce inflammatory responses, including cytokine secretion, NO production, and COX-2 expression in activated macrophages [56,57,58]. Since inflammation is considered to play a key role in the progression of NAFLD from steatohepatitis to cirrhosis and cancer [59], the anti-inflammatory effects of GSEE treatment appear to contribute to hepatoprotection against HFD.

The adipose tissue is the primary site for the storage of excess energy in the form of TG, which prevents lipotoxicity from ectopic lipid accumulation in other organs including liver [43,44]. In obesity, hypertrophy/hyperplasia of the WAT and reductions in brown adipose tissue (BAT) activity and abundance have been observed [36,51]. The activation of the BAT and the browning of the WAT which increase energy expenditure by heat production have been suggested to be promising strategies for improving obesity-associated metabolic diseases such as NAFLD [44,50]. In the present study, GSEE administration prevented HFD-induced adipose lipid accumulation by inhibiting white adipocyte differentiation and de novo lipogenesis as well as activating WAT browning (Figure 9). Similar anti-adipogenic and browning effects of an extract from germinated soy germ in HFD-fed obese mice were already reported in a previous study [27]. However, that study focused only on its effects on the adipose tissue and not on the liver and interrelation between the two organs.

It has been reported that there is direct crosstalk between the lipid metabolism of the liver and adipose tissue. For instance, adiponectin secreted by the adipose tissue is known to promote hepatic fatty acid β-oxidation, improve insulin signaling, and inhibit inflammation [60,61]. Moreover, studies have observed a reduction in circulating adiponectin levels and downregulation of hepatic adiponectin receptor levels in NAFLD [62,63]. Previously, adiponectin expression in adipose tissue, which was reduced in HFD-fed mice, was shown to be upregulated by GSEE treatment [27]. In obesity, activated WAT and the resident macrophages secrete inflammatory cytokines, such as TNF-α, IL-6, and IL-1β into the bloodstream, which stimulates hepatocytes and Kupffer cells [44,64]. Specifically, TNF-α reduces the insulin sensitivity of adipocytes, resulting in lipolysis, while the consequent release of FFA can adversely affect the liver [65,66]. On the contrary, an increase in the activity and mass of the BAT alleviates the progression of NAFLD in rodents by reducing the blood FFA levels [67,68]. In the present study, while it is unclear whether the adipose tissue or the liver was the primary target of GSEE, we demonstrated that GSEE affected the lipid metabolism of both the liver and adipose tissue and improved their physiological profiles. Considering the crosstalk between the liver and adipose tissue, the inhibition of WAT and upregulation of BAT by GSEE treatment could reduce the FFA load to the liver, which prevented ectopic lipid accumulation in hepatocytes. Conversely, the stimulation of hepatic lipid export induced by GSEE could also contribute to alleviating adipocyte fat accumulation, as an increase in the fatty acid flux from the liver was reported to suppress WAT lipogenesis [64]. Therefore, the dual activity of GSEE in both tissues could synergistically inhibit high-fat-promoted hepatotoxicity and adipogenesis, and thereby prevent obesity in mice.

Several molecular mechanisms explaining the hypolipidemic and hepatoprotective effects of soy ingredients have been suggested. Soy isoflavones, daidzein and genistein, have been shown estrogenic effects by binding to estrogen receptors (ER) since their chemical structures are similar with 17β-estradiol (E2) [8]. Through this ER-mediated pathway, soy isoflavones have shown anti-adipogenic activities by downregulating SREBP-1, PPARγ, and C/EBPα expression [69,70,71]. Not only soy isoflavones, but also soyasaponins have been shown to have antioxidant effects due to their radical scavenging activities against superoxide and hydrogen peroxide [72,73,74,75]. Moreover, soyasaponin Bb was reported to suppress NADPH oxidase-dependent reactive oxygen species (ROS) generation [76] and induce heme oxygenase-1 expression [19]. Oxidative stress is a critical player in the pathogenesis of NAFLD [1]. Thus, the estrogenic and antioxidant properties of soy compounds could be a possible explanation of the beneficial effects of GSEE. However, the exact mechanisms underlying its effects require further studies.

Efforts have been made to enhance the nutritional quality of soy-related products in order to improve their beneficial effects on human health. Moreover, to identify their exact mechanisms of action, extracted, isolated, or enriched ingredients have been used in both clinical and animal studies [6,8]. Soy embryos are abundant in soyasaponins and isoflavones when compared to other parts of the soybean, seed coat and cotyledon [20,21,22,23], but have been discarded due to their bitter taste. Despite their hepatoprotective effects [24], the therapeutic use of soy embryos can be limited due to their tiny size. Recent studies have shown that the germination process of soy embryos can markedly enrich the amount of bioactive substances in the germ [25,26,27]. The soyasaponin Ab concentration in the extract of germinated soybean embryos was shown to be 35.1 and 1.5 times higher than that in the whole soy and embryo, respectively [26,27]. After 24 h of germination, the concentrations of soy isoflavones, namely genistein, daidzein, and glycitein, were found to be increased by 39.1%, 21.4% and 31.1%, respectively [25]. Moreover, elevated tocopherols and free amino acid levels, as well as reduced concentrations of total carbohydrates, such as fructose and sucrose, in the germinated embryo suggest that germination might potentiate the hypolipidemic effect of soy embryos [25]. Moreover, the increased length, width, and thickness of the germinated embryo would enable the bulk production of soy germ-related products. In the present study, we also observed that the GSEE contains high concentration of soyasaponin Ab (Figure 1). Taken together, our results indicate that due to the hepatoprotective effect of GSEE against HFD, this extract can be used to develop effective medications for the treatment of NAFLD. 

## 4. Materials and Methods

### 4.1. Preparation of the Germinated Soybean Embryo Extract (GSEE)

The embryos of soybeans (*Glycine max* (L.) *Merr.*) cultivated in Jeongseon (Korea) were separated from the seed coats and cotyledons and germinated underwater in a stirring bath at 20 °C for 24 h. The soybean germ was then extracted using 50% ethanol (food grade) at room temperature for 6 h and filtered through a 75 μm cartridge. The GSEE powder was obtained by vacuum evaporation and spray drying. After quantification of the bioactive compound, soyasaponin Ab, by using an HPLC/MS/MS system, the extract was solubilized in distilled water (JW Pharmaceutical Co., Ltd., Seoul, Korea) and then used for further experiments.

### 4.2. Quantification of the Soyasaponin Ab in the GSEE by HPLC/MS/MS

A soyasaponin Ab standard (CAS No. 118194-13-1, Cat. No. BP1735, Chengdu Biopurify Phytochemicals Ltd., Chengdu, China) and the GSEE dissolved in 50% acetonitrile were injected into an HPLC system (Nexera X2, Shimadzu Co., Kyoto, Japan) connected to an Acquity C18 column (1.7 µm, 100 mm × 2.1 mm, Waters, Milford, MA, USA). We used mobile phase A (0.1% formic acid) and B (0.1% formic acid in acetonitrile) at a flow rate of 0.3 mL/min, and the gradient of mobile phase A was changed as follows: 0–3 min (95%), 10–12 min (0%), 12.1–15 min (95%). The peaks were detected by using the LCMS-850 (Shimadzu Co., Kyoto, Japan) system in the multiple reaction monitoring (MRM) mode. The mass spectra were monitored in the negative ionization mode, and the interface voltage was 4.0 kV. The retention time of the soyasaponin Ab peak was 9.157 min, and the ion masses of the precursor and products were 1435.9 and 221.3/262.9/161.2, respectively.

### 4.3. Animal Experiments

Male C57BL/6J mice (13-week-old), which were fed with control (10% of calories from fat) or HFD (60% of calories from fat) chow since they were 6-weeks old were purchased from Orient Bio Co., Ltd. (Seongnam, Korea). The animal experiments were carried out by Biotoxtech Co., Ltd. (Cheongju, Korea), and the protocols (No. 180273) were approved by the animal care and use committee of Biotoxtech Co., Ltd. The animal facility was maintained at a room temperature of 22 ± 2 °C and a humidity of 47 ± 17%, with a 12 h light–dark cycle. During the 2 weeks of acclimatization and 10 weeks of the experimental period, the mice were also provided with control 10% fat diet (CON, *n* = 8, energy density = 3.82 kcal/g, Cat. No. D12450J, Research Diet Inc., New Brunswick, NJ, USA) or 60% HFD chow (*n* = 40, energy density = 5.21 kcal/g, Cat. No. D12492, Research Diet Inc.) ad libitum. Mice in the HFD group (*n* = 24) were randomly divided into 3 groups (*n* = 8 in each group) and treated with GSEE (0, 15, or 45 mg/10 mL/kg of body weight orally) solubilized in distilled water (JW Pharmaceutical Co., Ltd.,) once a day for 10 weeks. Next, the mice were anesthetized via isoflurane inhalation, and blood samples were obtained via the abdominal vein. The liver and adipose (perirenal and epididymal) tissue were collected and stored in 10% neutral-buffered formalin, optimal cutting temperature (OCT) compound or a deep freezer (−80 °C). 

### 4.4. Serum Biochemical Analysis

The serum levels of total cholesterol (TC), triglycerides (TG), high-density lipoproteins (HDL) and low-density lipoproteins (LDL), as well as alanine transaminase (ALT) and aspartate transaminase (AST) activities, were measured using the Automated Chemistry Analyzer (Prestige 24i, Tokyo Boeki Medisys Inc., Tokyo, Japan). Free fatty acid (FFA) levels were determined using the Free Fatty Acid Assay Kit (BM-FFA-100, Biomax Co., Ltd., Seoul, Korea).

### 4.5. Histological Examinations of the Mouse Liver Tissue

For hematoxylin & eosin (H&E) staining, the liver tissues fixed in neutral-buffered formalin solution were embedded into paraffin. Then, the sectioned tissues (3–5 μm) were mounted on glass slides and stained with H&E. To examine hepatic lipid accumulation, the liver tissues embedded in the OCT compound were sectioned (10 μm) and fixed with 10% neutral-buffered formalin on a glass slide. The lipid droplets were stained with Oil red O, and the nuclei were stained using a hematoxylin solution. Histopathological examinations were performed using an inverted microscope (Olympus IX71, Olympus Co., Tokyo, Japan) equipped with a camera. Relative oil red O intensity in the microscopic images was measured by using ImageJ (National Institutes of Health, Bethesda, MD, USA).

### 4.6. Hepatic Lipid Quantification

The liver homogenate was used to determine of TG and FFA levels. Hepatic lipids were extracted by chloroform and methanol, and the TG concentration in the organic phase was measured using a TG Assay Kit (AM157S-K, Asan Pharmaceutical, Seoul, Korea). Hepatic FFA levels were quantified using a Free Fatty Acid Assay Kit (BM-FFA-100, Biomax Co., Ltd.).

### 4.7. Real-Time Reverse Transcription Polymerase Chain Reaction (qRT-PCR)

Liver and adipose tissue from the experimental mice were used for qRT-PCR. Total RNA isolation and cDNA synthesis were conducted using the Direct-zol™ RNA MiniPrep (Zymo Research, Irvine, CA, USA) and iScript cDNA synthesis kit (Bio-Rad Laboratories, Inc., Hercules, CA, USA), respectively. The qRT-PCR was carried out using the SensiFAST™ SYBR^®^ No-ROX Kit (Bioline, London, UK) on the CFX Connect^TM^ Real-Time System (Bio-Rad Laboratories, Inc.). The primers used in this study are listed as follows (Table 1).

### 4.8. Western Blotting

Liver tissue was lysed in RIPA buffer, and the centrifuged (10,000× *g*, 4 °C, for 20 min) supernatant was used for the determination of total protein content by using a BCA protein assay kit (Thermo Scientific, Sunnyvale, CA, USA). The equal amounts of proteins were separated by SDS-PAGE, and transferred onto nitrocellulose membrane (Bio-Rad Laboratories, Inc., Hercules, CA, USA). The membranes were blocked with 5% non-fat powdered milk at room temperature for 1 h, and incubated with the following specific primary antibodies (1:2000 to 1:5000 dilution) overnight at 4 °C: SREBP-1 (sc-8984, Santa Cruz Biotechnology, Inc., Dallas, TX, USA), AMPKα (5831, Cell Signaling Technology, Inc., Danvers, MA, USA), p^Thr172^-AMPKα (2535, Cell Signaling Technology, Inc.), PPAR-α (ab24509, Abcam, Cambridge, UK), and GAPDH (sc-32233, Santa Cruz Biotechnology, Inc.). After washing, the membranes were incubated with the appropriate horseradish peroxidase-conjugated secondary antibodies for 1 h, and the antigen–antibody complexes were detected using an EZ-Western Lumi Pico detection kit (Dogen, Seoul, Korea).

### 4.9. Statistical Analysis

All results are expressed as mean ± standard error (SE). The results were analyzed using two-tailed unpaired Student’s *t*-test. *p*-value of < 0.05 was considered statistically significant.

## 5. Conclusions

Herein, we document for the first time the hepatoprotective effect of GSEE in HFD-fed obese mice. We found that GSEE alleviated hepatic injury, lipid accumulation, and inflammation by modulating the related gene expressions and protein levels. Activation of AMPK, which inhibits lipogenesis and stimulates FA oxidation, can be suggested to be an important mechanism of the anti-steatogenic effect of GSEE. This extract also reduced obesity via suppressing adipogenesis in adipose tissue, which appeared to beneficially affect hepatic lipid metabolism by lowering circulating lipid levels. Although soyasaponin Ab was present in abundant quantities in the GSEE, it is still unclear what soy component is the major contributor to the beneficial effects of the GSEE. Additionally, a direct comparison between the efficacy of whole soy, embryo, and germinated embryo extracts against hepatic steatosis has not been performed. Nevertheless, the results of this study suggest that dietary intake of germinated soy embryos and supplements containing the extract can be promising strategies for the prevention and treatment of NAFLD. In particular, our findings provide the possibility that the process of germinating embryos could improve the beneficial function of soybeans against chronic liver disease as well as obesity.

## Figures and Tables

**Table 1:** List of primers used for qRT-PCR.

Genes	Primer Sequences	
*ACOX*	F: GGAAGACTTCCAATCATGCGATAG	R: GACAACAAAGGCATGTAACCCG
*ApoB*	F: TTGGCAAACTGCATAGCATCC	R: TCAAATTGGGACTCTCCTTTAGC
*C/EBPα*	F: CAAGAACAGCAACGAGTACCG	R: GTCACTGGTCAACTCCAGCAC
*CD14*	F: AAACTCGCTCAATCTGTCTTTCACT	R: TCCTATCCAGCCTGTTGTAACTGA
*CD36*	F: CCTTGGCAACCAACCACAAA	R: ATCCACCAGTTGCTCCACAC
*CIDEA*	F: TGACATTCATGGGATTGCAGAC	R: CATGGTTTGAAACTCGAAAAGGG
*COX2*	F: GCATTCTTTGCCCAGCACTT	R: AGACCAGGCACCAGACCAAAG
*F4/80*	F: GTGACTCACCTTGTGGTCCT	R: CAGACACTCATCAACATCTGCG
*FAS*	F: AGGTGGTGATAGCCGGTATGT	R: TGGGTAATCCATAGAGCCCAG
*IL1β*	F: TTCACCATGGAATCCGTGTC	R: GTCTTGGCCGAGGACTAAGG
*IL6*	F: TTGCCTTCTTGGGACTGATG	R: CCACGATTTCCCAGAGAACA
*iNOS*	F: CGAAACGCTTCACTTCCAA	R: TGAGCCTATATTGCTGTGGCT
*MTTP*	F: CTCTTGGCAGTGCTTTTTCTCT	R: GAGCTTGTATAGCCGCTCATT
*PGC1α*	F: TATGGAGTGACATAGAGTGTGCT	R: CCACTTCAATCCACCCAGAAAG
*PPARγ*	F: GGAAGACCACTCGCATTCCTT	R: GTAATCAGCAACCATTGGGTC
*PRDM16*	F: CCACCAGCGAGGACTTCAC	R: GGAGGACTCTCGTAGCTCGAA
*SREBP1c*	F: GATGTGCGAACTGGACACAG	R: CATAGGGGGCGTCAAACAG
*TNFα*	F: GGCCTCTCTACCTTGTTGCC	R: CAGCCTGGTCACCAAATCAG
*UCP1*	F: AGCCATCTGCATGGGATCAAA	R: GGGTCGTCCCTTTCCAAAGTG
*β-actin*	F: ACGTCGACATCCGCAAAGACCTC	R: TGATCTCCTTCTGCATCCGGTCA
